# A Novel ARTP-derived *Bacillus megaterium* Mutant with Enhanced Salt Tolerance and Plant Growth Promotion in Saline–alkali Soil

**DOI:** 10.1007/s00284-026-05077-9

**Published:** 2026-08-02

**Authors:** Min Sun, PengFei Qiu, XinJie Yuan, QiLi Zhu, ZhenXing Peng, JiMin Lv, HongShun Li, JunPing Bao, YanQing Wei, ShuDong Qu, XianShun Ren, ZiHao Wang, Yi Ding, YongJun Wu, Wei Liu

**Affiliations:** 1 Syngenta Group China, Sinofert Holdings Limited, Beijing, 100069 China; 2 National Engineering Research Center for Cultivated Land Protection, Sinochem Agriculture Linyi R&D Center, Linyi, 276024 China; 3 National Key Laboratory of Biochemical Engineering, Key Laboratory of Biopharmaceutical Preparation and Delivery, Institute of Process Engineering, Chinese Academy of Sciences, Beijing, 100190 China; 4 College of Life Sciences, Northwest A&F University, Yangling, 712100 China

## Abstract

Soil salinization seriously threatens global food security with continuously expanding affected areas. *Bacillus megaterium*-based bioinoculant serves as an eco-friendly strategy for saline–alkali land remediation, whereas wild-type (WT) strains generally exhibit poor salt–alkali tolerance. To address this limitation, atmospheric and room temperature plasma (ARTP) mutagenesis combined with a microbial microdroplet culture (MMC) high-throughput screening system was adopted to screen high salt-tolerant *B. megaterium* mutant followed by multi-omics characterization. Using WT *B. megaterium* LD (CGMCC No.21828) as the original strain, a mutant library was constructed via optimized ARTP treatment, and the elite mutant SLD48 was isolated under dynamically increasing salt gradients. Compared with the wild type, SLD48 possesses a larger genome of 6,252,538 bp and acquires 28 extra functional genes including *spoIVCA* and *pJ_gene0003*. Intracellular osmoprotectants (proline, glycine betaine) and antioxidant α-tocopherol were markedly accumulated in SLD48. moreover, exogenous supplementation of these substances in the culture medium can effectively alleviate the damage caused by salt stress to the cells. In pot trials with saline–alkali soil, the rhizosphere colonization of SLD48 reached 3.52 × 10⁵ CFU/g (2.46-fold of LD), and maize shoot fresh weight increased by 40.5% after inoculation. Collectively, this work confirms that mutant SLD48 is a promising microbial resource for saline–alkali soil improvement and crop yield promotion.

**Supplementary Information:**

The online version contains supplementary material available at https://doi.org/10.1007/s00284-026-05077-9.

## Introduction

With the exacerbating related environmental issues, the uncontrolled use of chemical fertilizers, climate change, and seawater intrusion, soil salinization has intensified and now threatens global food production [1]. According to statistics from the Food and Agriculture Organization of the United Nations (FAO), more than 1 billion hectares of land are affected by salinization, and this area is expanding at an annual rate of 1.5%. The resulting decline in crop productivity leads to an annual economic loss of ~ 27.3 billion US dollars. In China, ~ 36 million hectares of cultivated land in the Huang-Huai-Hai Plain and Northeast regions are salinized, causing a 30–50% yield reduction in major crops [2]. Typical salts in soil and water include sodium chloride, calcium chloride, calcium sulfate, potassium chloride, etc. [3]. Sodium chloride and magnesium chloride are the predominant components in saline-alkali soils, and chloride ions are toxic to plants because high concentrations inhibit growth [4]. As a result, developing agricultural practices suitable for saline-alkali land has become a promising approach to strengthen global food security. Microbial improvement technology has recently attracted considerable attention as a sustainable and environmentally friendly strategy for ameliorating saline-alkali soils. Microorganisms can alleviate salt stress through several mechanisms: (1) synthesizing osmolytes such as proline and betaine to maintain cellular osmotic balance, (2) secreting organic acids such as citric and oxalic acids to promote mineral weathering, and (3) regulating rhizosphere micro ecology to enhance plant salt tolerance [5, 6].

*Bacillus megaterium* is a promising candidate strain for saline–alkali soil remediation due to its strong environmental adaptability and diverse metabolic capacity. It enhances plant tolerance by secreting polysaccharides and organic acids, and it produces phytase that mobilizes insoluble phosphorus, thereby simultaneously reducing soil salinity and improving fertility [7–9]. Additionally, it interacts synergistically with arbuscular mycorrhizal fungi to strengthen their functions [10]. Previous research demonstrated that applying *B. megaterium* improved the physio-biochemical and anatomical characteristics of wheat seedlings grown under saline conditions [11].

However, the tolerance of wild-type (WT) *B. megaterium* to saline-alkali conditions is inherently limited. In non-halophilic bacteria, normal growth typically occurs only at salt concentrations below 1% [12]. So, in this study, we applied mutagenesis and salt-tolerance acclimatization to existing halo-tolerant strains to enhance their performance.

Random mutagenesis, a non-transgenic breeding strategy, is widely employed to generate microbial mutants. Among chemical and physical mutagenesis approaches, atmospheric and room temperature plasma (ARTP) is a particularly effective physical mutagenesis method frequently used to construct microbial mutant libraries [13, 14]. ARTP generates high-density reactive chemical species that damage DNA and induce mutations, thereby modifying microbial metabolic profiles. The Microbial Microdroplet Culture system (MMC) [13] further provides an efficient platform for screening salt-tolerant mutants.

## Materials and Methods

### Microorganisms and Seed Liquid Cultivation

*B. megaterium* LD, the WT strain isolated from maize rhizosphere soil and deposited in the China General Microbiological Culture Collection Center (CGMCC No. 2182821 [15]), was cultured in nutrient broth (NB) containing (per liter): peptone 5 g, beef extract 3 g, NaCl 5 g, pH 7.0–7.2 at 37℃ and 200 rpm 12 h for activation [15].

The activated bacterial suspension was inoculated into fresh NA medium at a 3% inoculum rate and cultured until the logarithmic phase (OD600 = 0.6–0.8). Cells were collected by centrifugation at 4000 rpm for 10 min, washed three times with 85% sterile saline, and resuspended in NA medium. The OD600 was then adjusted to 1 and diluted 100-fold to prepare the seed solution for later use.

### ARTP Mutagenesis

The ARTP mutagenesis procedure followed established protocols [16]. Briefly, 10 µL of the seed solution (10⁶–10⁸ CFU/mL) was evenly spread on a stainless-steel slide and placed into the ARTP chamber (Wuxi Yuanqing Tianmu Biotechnology Co., Ltd.). Ultrapure helium served as the plasma working gas, with parameters set as follows: radio frequency power of 120 W, helium flow rate of 10 L/min, and a 2 mm distance between the plasma torch nozzle and the sample plate.

Modern breeding theory indicates that when mutagenesis-induced mortality in microorganisms exceeds 95%, the positive mutation rate reaches its peak and mutagenic efficiency is optimal [17, 18]. Therefore, to increase the likelihood of isolating strains with enhanced viability and protein synthesis capacity, the cell suspension was uniformly spread on a sterile metal plate and subjected to plasma irradiation for 0–600 s. The irradiation duration producing 95% mortality was selected as the optimal treatment time.

After ARTP treatment, the stainless-steel slide was transferred to a 2 mL centrifuge tube containing 1 mL sterile water and vortexes to elute the cells. A 100 µL aliquot of the diluted suspension was spread on NA solid medium (1.5% agar) and incubated at 32℃ for 12 h before colony counting. Mortality was calculated as: Mortality rate (%) = [(Number of control colonies − Number of treatment colonies) / Number of control colonies] × 100%.

### Single-factor, Multi-level Experiment on WT *B. megaterium* LD

High-throughput droplet culture was conducted using the MMC-B1 microbial culture system (Wuxi Yuanqing Tianmu Biotechnology Co., Ltd.). Eight salt concentration gradients were automatically generated using the “single-factor, multi-level” function: 5.6‰, 5.98‰, 6.41‰, 6.79‰, 7.225‰, 7.6‰, 7.98‰, and 9.6‰. A mixed NaCl and MgCl₂ solution (1:1, mass ratio) was used [19].

During each operation, droplets containing the WT strain were cultured under the different salinity conditions. Growth curves were monitored in real time using OD_600_. Based on these observations, the salt concentration that inhibited the growth of the WT strain LD was selected as the baseline salinity for subsequent mutant screening.

### Screening of *B. megaterium* Mutants

The mutant strains obtained after ARTP mutagenesis were inoculated into shake-flask medium and cultured to the logarithmic growth phase. The bacterial suspension was then adjusted to 1 × 10⁶–1 × 10⁸ CFU/mL. A 5% inoculum of each mutant strain was transferred into fresh fermentation medium (containing 5% (w/v) soybean meal, 6% corn flour, 1.5% starch, 0.5% glucose, 0.2 g/L MnSO₄, 3 g/L KH₂PO₄; final pH 7.5), mixed thoroughly, and loaded into the MMC-B1 system for screening.

The screening parameters were set as follows: 200 droplets (numbered 1 ~ 200) and a detection wavelength of 600 nm. Based on the growth profile of the WT strain LD, the MMC system automatically generated salt concentrations of 5.6‰, 5.98‰, 6.41‰, 6.79‰, 7.225‰, 7.6‰, 7.98‰, and 9.6‰ for mutant acclimation. As salt levels increased, five mutants exhibiting superior growth were selected.

### Identification and Analysis of *B. megaterium* Mutants

Morphological characteristics of *B. megaterium* mutants were examined using a stereomicroscope and a scanning electron microscope (SEM). Single colonies were first obtained by inoculating the mutants onto solid Luria–Bertani (LB) medium [15]. Colony morphology, size, and surface features were observed using a stereomicroscope (OLYMPUS SZ61).

**For SEM analysis**, mutants were cultured in liquid LB medium to the logarithmic phase (typically 12–16 h). A 2 mL centrifuge tube was used to collect the culture, which was centrifuged at 8000 rpm for 10 min. After discarding the supernatant, 2 mL of 2.5% glutaraldehyde was added along the tube wall to avoid disturbing the pellet, and the sample was fixed overnight at 4 °C. The pellet was rinsed with 1.5 mL of 0.1 M phosphate buffer, gently mixed by pipetting, and centrifuged at 8000 rpm for 10 min. After discarding the supernatant following buffer removal, dehydration was performed with 1.5 mL ethanol at 30%, 50%, 70%, 80%, and 100%, with 100% ethanol applied twice. Each step lasted 10 min, and after each addition, the sample was gently mixed and centrifuged at 8000 rpm for 10 min. The pellet was then rinsed once with 1.5 mL of 50% isopentyl acetate and twice with 1.5 mL of 100% isopentyl acetate (Tianjin BASF Chemical Co., Ltd.), with centrifugation at 8000 rpm for 10 min after each rinse. The bacterial cells were dried using an XD-1 carbon dioxide critical-point dryer (Eiko) and sputter-coated with gold using an IB-3 ion coater (Eiko). SEM imaging was performed with a JSM-840 scanning electron microscope (JEOL).

**For whole genome sequencing**, the WT strain LD and two mutant strains were analysed (1 biological replicate for each line, the whole-genome sequences have been deposited in the NCBI GenBank database under BioProject ID PRJNA1469293). Draft genome sequencing was conducted on the Illumina platform. Shanghai Majorbio Bio-pharm Technology Co., Ltd. performed deep sequencing of genomic DNA using a combined PacBio Sequel IIe and Illumina approach.

**For metabolite extraction**, 100 µL of bacterial suspension was mixed with 400 µL of extraction solution (acetonitrile: methanol = 1:1, containing 0.02 mg/mL L-2-chlorophenylalanine as the internal standard). After vertexing for 30 s, samples underwent low-temperature ultrasonic extraction for 30 min (5℃, 40 kHz) and were then placed at -20℃ for 30 min. Samples were centrifuged at 13,000 g for 15 min at 4℃, and the supernatant was collected, dried under nitrogen, and reconstituted with 100 µL of reconstitution solution (acetonitrile: water = 1:1). Following a 5 min ultrasonic extraction (5℃, 40 kHz), samples were centrifuged again at 13,000 g for 10 min at 4℃, and the resulting supernatant was used for analysis (3 biological replicates).

LC-MS/MS analysis was performed by Shanghai Majorbio Biomedical Technology Co., Ltd. After data acquisition, raw LC-MS files were processed using Progenesis QI. MS and MS/MS spectra were matched against the HMDB (http://www.hmdb.ca/) and Metlin (https://metlin.scripps.edu/) databases to identify metabolites.

In addition to genome and metabolomic analyses, we assessed the colonization ability of mutant strains in saline-alkali soil. Maize served as the model plant, and four treatments were established: CK, *B. megaterium* WT strain LD, mutant strain SLD14, and mutant strain SLD48. Each pot contained 7 kg of saline-alkali soil with a salt content of 4.24 g/kg and one maize plant, with 15 replicates per treatment. Plant roots were irrigated with bacterial suspensions to ensure 3 million CFU/g viable cells in the soil; the CK group received water only. Plants were cultivated in a greenhouse at 25 °C with a relative humidity of 70% under a 16 h light / 8 h dark photoperiod. After one month of culture, the rhizosphere colonized viable cell counts and maize growth parameters were determined.

### Salt Tolerance Evaluation of Strains

#### Preparation of Crude Bacterial Enzyme Extract

Bacterial cells were disrupted using a combination of lysozyme lysis and repeated freeze–thaw cycles. Lysis buffer was prepared as follows: 6.06 g Tris-HCl, 8.77 g NaCl, 0.372 g EDTA-Na₂·2 H₂O, and 0.0087 g PMSF were dissolved in sterile deionized water to a final volume of 100 mL, and the pH was adjusted to 8.0. Before use, 100 mg lysozyme was added and mixed thoroughly.

Bacterial cultures at the logarithmic growth phase were centrifuged to collect cells. The cell pellet was resuspended in the prepared lysis buffer and incubated in a water bath at 37 ℃ for 30 min. The suspension was then frozen at − 20 ℃ for 30 min, thawed in a 37 ℃ water bath, and the freeze–thaw cycle was repeated 2–3 times. After centrifugation at 12,000 rpm for 15 min, the supernatant was collected as the crude bacterial enzyme extract and stored on ice.

#### Determination of Antioxidant and Osmotic Adjustment Indices

Activities of catalase (CAT) and superoxide dismutase (SOD), as well as contents of malondialdehyde (MDA) and proline (PRO), were determined using commercial kits from Bioswanp (BTK021, BTK032, BTK022, and BTK082, respectively) following the manufacturer’s instructions.

#### Determination of Cell Membrane Conductivity

Bacterial suspensions cultured under different NaCl concentrations for 48 h were adjusted to the same OD₆₀₀ value. The conductivity was directly measured using a bench-top conductivity meter (HANNA instruments HI 2315). Each treatment was performed in triplicate.

### Influence of Osmoprotectants on Strain Growth at 8‰ NaCl

A salt stress condition was set using an 8‰ NaCl. Proline (Pro, 32 mmol/L), α-tocopherol (α-Toc, 150 µmol/L), betaine (Bet, 15 mmol/L), 5-aminovaleric acid (5-AVA, 3 mmol/L), and succinic acid (SuA, 3 mmol/L) were separately added to the medium [20–23]. Meanwhile, two control groups were established: a blank control without salt and metabolites (CK), and a salt stress control with only 8‰ salt.

Strains LD and SLD-48 were inoculated into each treatment medium at the same inoculum size. After incubation at an appropriate temperature, the OD₆₀₀ value and viable cell count of the bacterial suspension were determined to analyze the alleviating effects of different metabolites on strain growth under salt stress.

**Fig. 1:**
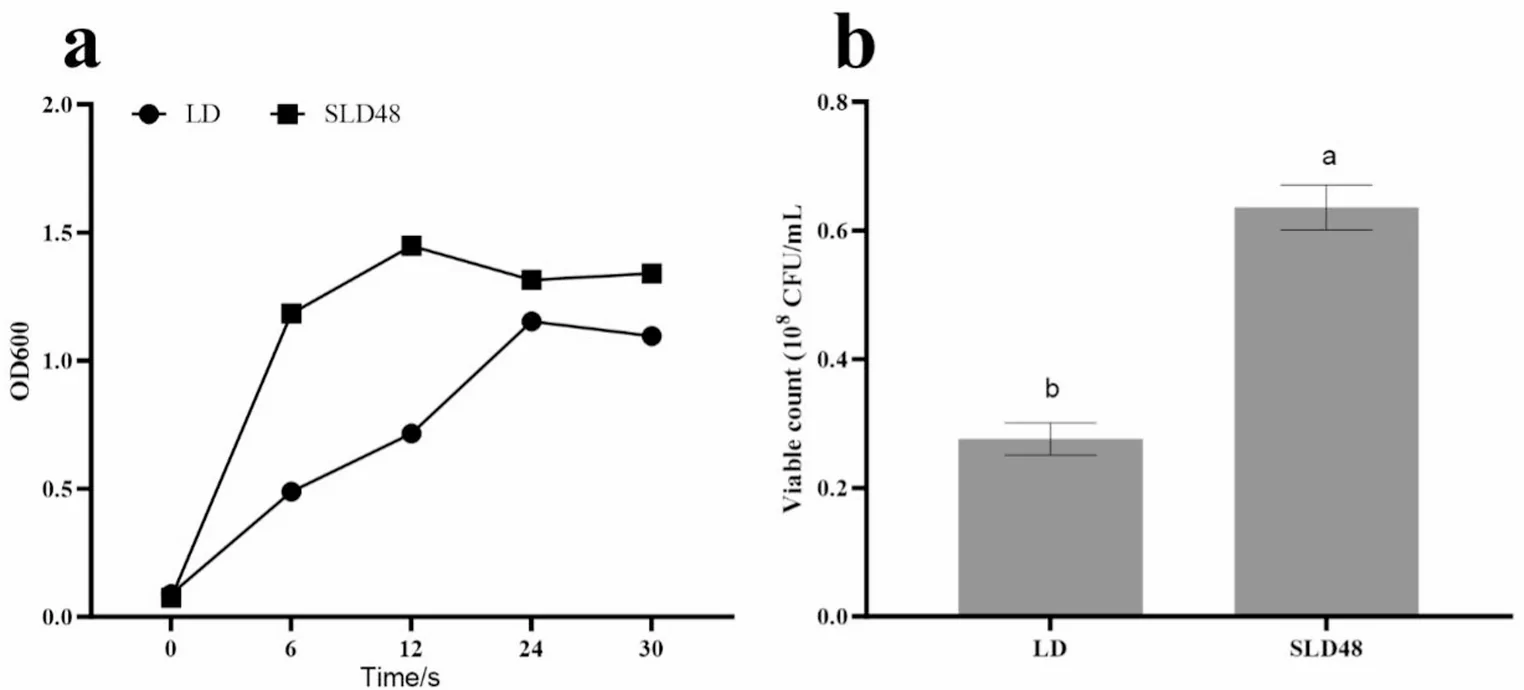
Growth rate and viable cell count of LD and SLD48 at a salt concentration of 8.5‰. **a** Growth rate of SLD48 and LD at a salt concentration of 8.5‰, **b** The viable cell count of the fermentation broth cultured for 30 h at a salt concentration of 8.5‰. The values are the mean of 3 repetitions. The error bars are the standard deviation. Values with the same letters do not have a significant difference by Student’s t-test (95%)

**Fig. 2:**
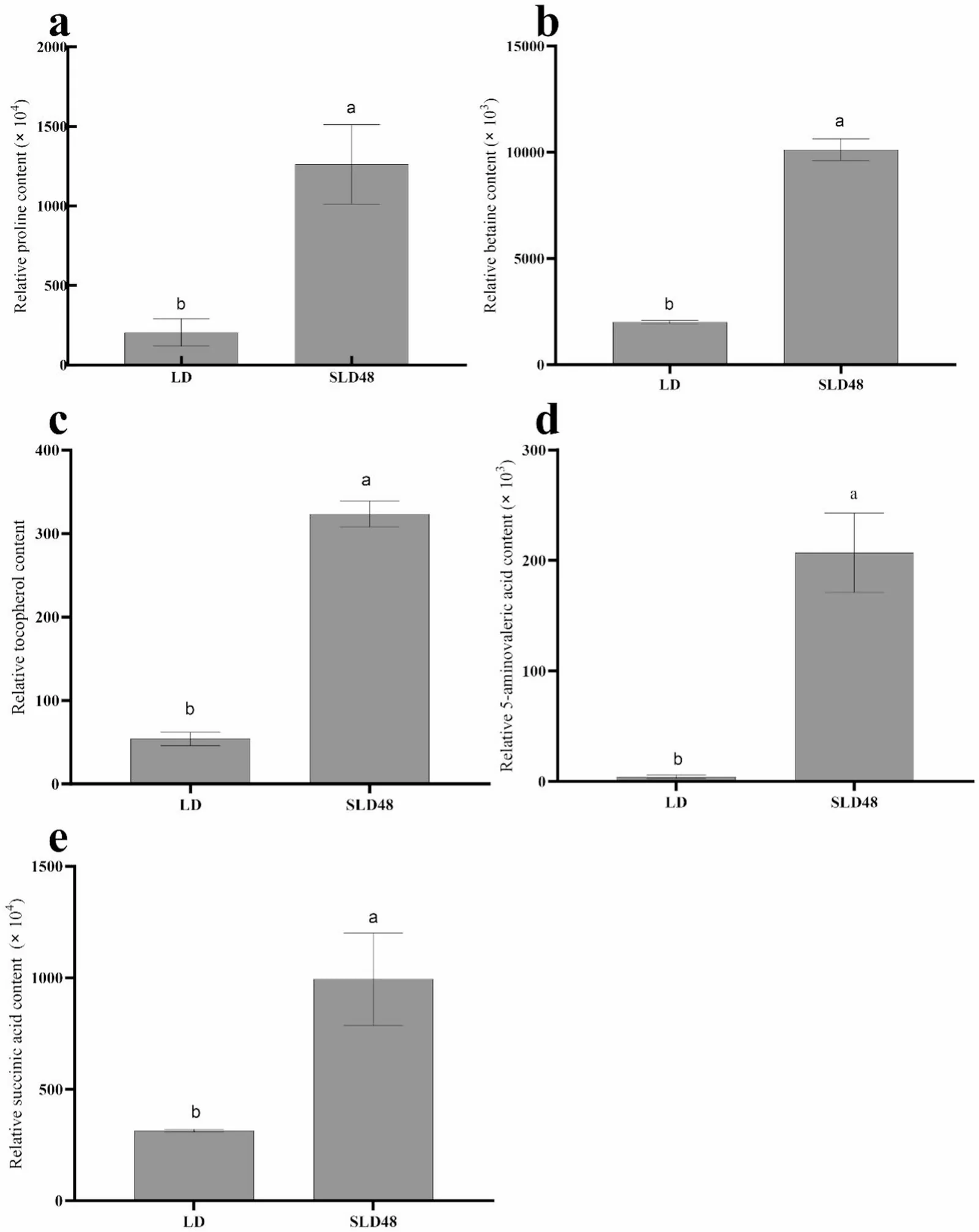
Relative metabolite content of LD and SLD48. **a** proline, **b** betaine, **c** tocopherol, **d** 5-aminovaleric acid, **e** succinic acid. The values are the mean of 3 repetitions. The error bars are the standard deviation. Values with the same letters do not have a significant difference by Student’s t-test (95%)

**Fig. 3:**
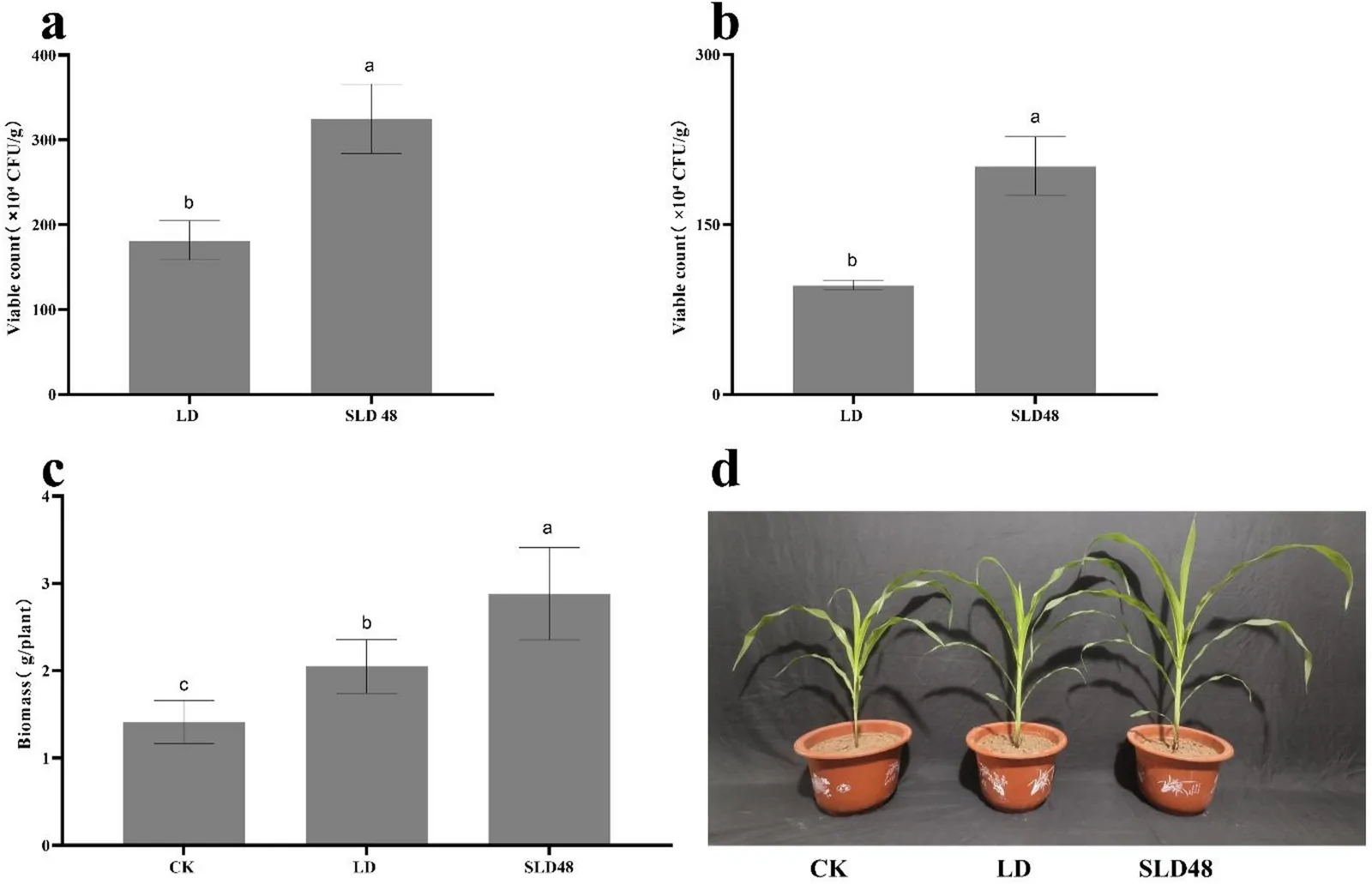
Salt tolerance evaluation of LD and SLD48 on maize in saline soil (NaCl 5‰). **a** Viable cell count at 24 h after inoculation, **b** Viable cell count at 30 days after inoculation, **c** Whole-plant biomass of maize at 40 days after inoculation, **d** Maize plants at 40 days after inoculation. The values are the mean of 3 repetitions. The error bars are the standard deviation. Values with the same letters do not have a significant difference by Student’s t-test (95%)

**Fig. 4:**
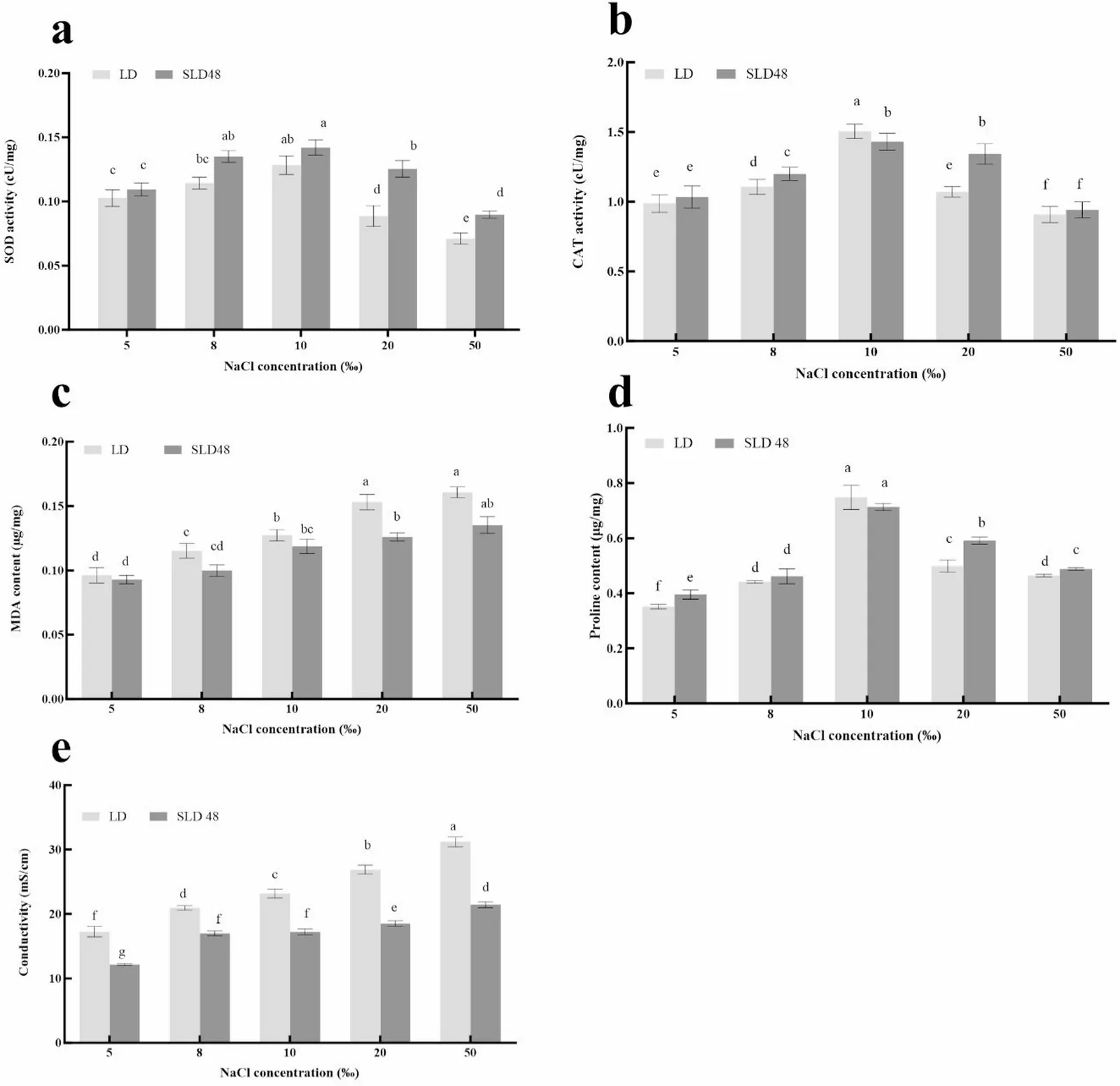
Antioxidant capacity and osmotic adjustment evaluation of LD and SLD48 under gradient salt concentrations. **a** SOD activity, **b** CAT activity, **c** MDA content, **d** proline content, **e** membrane electrical conductivity. The values are the mean of 3 repetitions. The error bars are the standard deviation. Values with the same letters do not have a significant difference by Tukey test (95%)

**Fig. 5:**
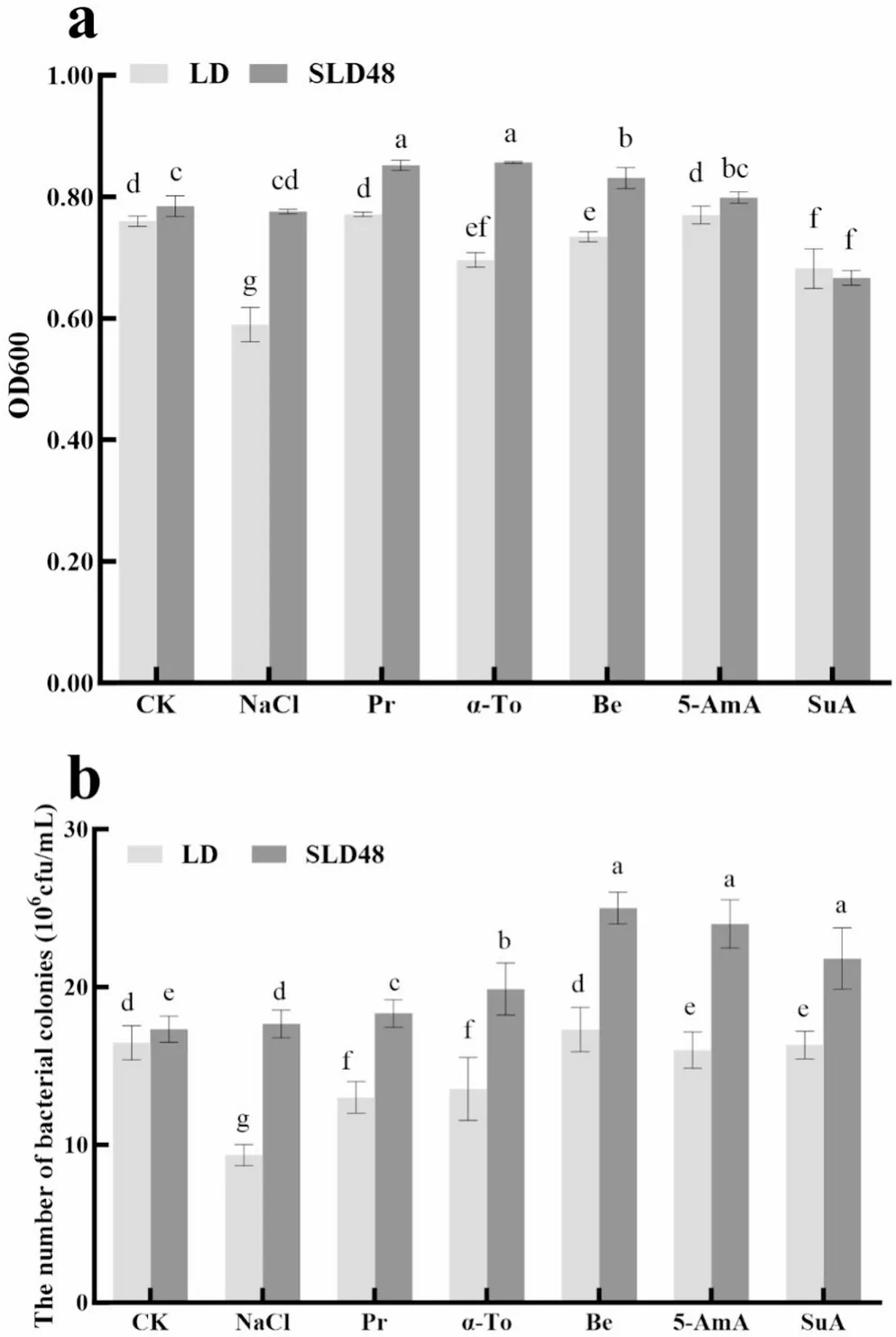
Growth performance of LD and SLD48 with adding metabolites under 8‰ NaCl stress. **a** OD₆₀₀ values, **b** viable cell count. The values are the mean of 3 repetitions. The error bars are the standard deviation. Values with the same letters do not have a significant difference by Tukey test (95%)

### Data Analysis

Principal component analysis (PCA) and orthogonal partial least squares discriminant analysis (OPLS-DA) were performed on the preprocessed data matrix using the ropls package (version 1.6.2) in R. Significantly differential metabolites were screened based on the variable importance in projection (VIP) values from the OPLS-DA model and p-values from Student’s *t*-test. Metabolites with *p* < 0.05 were regarded as significantly different. The differential metabolites were annotated and mapped to metabolic pathways using the KEGG PATHWAY database (https://www.kegg.jp/kegg/pathway.html). Pathway enrichment analysis was carried out using Python packages, and the enriched metabolic pathways were identified by Fisher’s exact test. Other statistical analyses were performed using SPSS 22.0, and differences were considered statistically significant at *p* < 0.05.

**Table 1:** Differences of whole-genome sequences of LD and SLD48

Item	LD	SLD48
Total genome size	6,228,701 bp	6,252,538 bp
Number of endogenous plasmids	9	11
GC content (%)	37.53	37.51
Number of functional genes	6332	6360
Number of tRNAs	145	145
Number of rRNAs	43	43
Number of sRNAs	116	117

**Table 2:** Unique functional genes of SLD48 compared with the WT strain

Gene ID	Gene name	Gene size	Gene description
pJ_gene0004	spoIVCA	801 bp	Recombinase family proteins
pJ_gene0003	—	711 bp	MerR family proteins
pJ_gene0009	—	1983 bp	MobA/MobL family proteins

## Results

### Screening of Mutant Strains

Based on the lethality curve of the WT *B. megaterium* LD during ARTP mutagenesis (Supplementary Fig. 1a), the optimal irradiation duration for ARTP mutagenesis was determined. When the lethality rate reached 85–95%, the corresponding ARTP exposure time was 25–35 s. Therefore, the bacterial suspension from this period was transferred into fresh medium to construct the mutant library.

The collected mutant library samples were screened using the MMC system. Mutant and WT bacterial suspensions were inoculated separately into fermentation media with serial salinity gradient concentrations to obtain the growth curves of the strains (Supplementary Fig. 1b). During the 40 h cultivation of the WT suspension, strain growth decreased progressively with increasing salinity. At 9.6‰, inhibition was strongest; at 6.79‰, growth at all time points was lower than that at 5.6‰ but did not cease. Therefore, 6.79‰ was selected as the initial salt concentration for mutant screening.

Library cultures were inoculated into fermentation medium at an inoculation ratio of 5% (v/v), fully homogenized, and loaded into the MMC system, which automatically generated a continuous salinity gradient spanning 6.79‰ to 8.5‰. Following successive subculturing, a single superior-growth mutant was isolated and named SLD48. The mutant SLD48 and parental strain LD were cultured independently under 8.5‰ salinity (Fig. 1a). All five candidate mutants exhibited markedly accelerated growth with identical growth trends; obvious elevation in OD₆₀₀ was detected for each mutant after 10 h of incubation.

Viable cell enumeration of 30 h fermentation broth was conducted using the spread-plate method [23]. As shown in Fig. 1b, the viable count of the WT strain LD was 2.76 × 10⁷ CFU/mL, whereas that of mutant strain SLD48 increased significantly to 6.36 × 10⁷ CFU/mL.

### Identification of Mutant Strain

The morphological characteristics of mutant SLD48 were observed under a stereomicroscope and scanning electron microscope (SEM). Compared with the WT (Supplementary Fig. 2a), colonies of SLD48 (Supplementary Fig. 2b) showed smoother surfaces with fewer wrinkles. SEM imaging further displayed a smoother cellular membrane for SLD48. These observations ARTP mutagenesis remodeled the biofilm structure of SLD48 (Supplementary Fig. 2c, d).

Whole-genome sequencing was performed on both strains (Supplementary Data 1,2) revealing prominent genomic structural variations between SLD48 and its parental WT LD. The genome of LD is 6,228,701 bp in length with a GC content of 37.53% (Table 1), whereas SLD48 has an enlarged genome of 6,252,538 bp, 11 endogenous plasmids and an additional 28 functional genes.

### Genomic and Metabolomic Analysis of Mutant Strain

Genomic analysis also revealed that SLD48 contains several genes absent in LD (Table 2). Among them, the RecA/Rad51 superfamily which has been reported as recombinase spoIVCA participates in repairing DNA double-strand breaks (DSBs) through homology-directed repair [24]. The pJ_gene0003 gene is recorded to encode a MerR family regulatory protein, and proteins of this family can act as transcriptional regulators mediating microbial heavy metal tolerance and detoxification [25]. MobA and MobL have been reported to encode conjugative plasmid transfer proteins [26, 27]. However, the roles of these genes in *Bacillus megaterium* still need to be verified by constructing specific mutant strains.

Metabolomic analyses further showed that SLD48 exhibits pronounced metabolic adjustments associated with salt stress adaptation (Fig. 2). Proline (Pro) [28, 29] and glycine betaine (GB) [30–32] which have been reported as osmoprotectants, significantly increased by 515% and 403%, respectively (Fig. 2a and b). α-Toc, a central component of the non-enzymatic reactive oxygen species (ROS) scavenging system [33–35], increased significantly, with levels in SLD48 497% higher than those in LD (*p* < 0.001) (Fig. 2c). The 5-aminovaleric acid (AVA) also significantly increased in SLD48 (Fig. 2d). We also observed a significant increase in succinic acid (SA, Fig. 2e), which has been reported to help strains overcome growth and metabolic limitations [36].

### Pot Experiments on Maize Salt Tolerance

Next, we evaluated the rhizosphere colonization ability and growth promotion of the mutant strain in corn rhizosphere soil under salt stress using pot experiments (Soil physicochemical properties are shown in Supplementary Table 1). The results showed (Fig. 3a, b) that application of a 3 × 10⁶ CFU/g bacterial suspension to the corn rhizosphere significantly increased the colonization density of SLD48 in saline soil (NaCl 5‰, EC = 4.8 mS/cm) to 2.09 times that of the WT strain LD (LD: 9.65 × 10⁵ CFU/g; SLD48: 2.02 × 10^6^ CFU/g, *P* < 0.01). Phenotypic measurements in 40 days further demonstrated (Fig. 3c) that shoot fresh weight in the SLD48 treatment increased by 40.5% compared with LD (LD: 2.05 g; SLD48: 2.88 g; *P* < 0.001). Together, these findings indicate that SLD48 enhances rhizosphere bacterial colonization efficiency by improving survival and resource competition under salt stress.

### Evaluation of Salt Tolerance of the Mutant Strain

To evaluate the salt tolerance of the WT strain LD and the mutant strain SLD48, the growth of the two strains was monitored continuously for 48 h under five salt concentration gradients (5‰, 8‰, 10‰, 20‰, and 50‰), which was characterized by the OD₆₀₀ value. Both strains exhibited a typical growth pattern: lag phase (2–8 h), logarithmic growth phase (8–30 h), and stationary phase (30–48 h) (Supplementary Fig. 3).

At salt concentrations of 5‰ and 8‰, SLD48 entered the logarithmic growth phase at approximately 8 h, which was 2 h earlier than LD. Moreover, the OD₆₀₀ value at the stationary phase was significantly higher than that of LD, indicating a faster growth rate.

Under moderate and high salt concentrations (10‰, 20‰, and 50‰), the growth of LD was severely inhibited with almost no obvious logarithmic phase and an extremely low OD₆₀₀ value at the stationary phase (0.130–0.350). In contrast, SLD48 maintained a complete growth cycle: the OD₆₀₀ value at the stationary phase reached 0.995 at 10‰ salt, and remained at 0.393 and 0.200 at 20‰ and 50‰, respectively, demonstrating a higher salt tolerance threshold.

### Antioxidant Capacity and Osmotic Adjustment Evaluation

To elucidate the enhanced antioxidant capacity of SLD48 under salt stress, the activities of SOD and CAT, as well as MDA content, were determined in LD and SLD48 under gradient salt stress (Fig. 4).

The SOD activity of SLD48 was higher than that of LD at all tested salt concentrations, and the difference was extremely significant (*P* < 0.01, Fig. 4a) in the range of 20‰–50‰ salt. The CAT activity of SLD48 was significantly higher than that of LD at 5‰ and 20‰ salt. Correspondingly, the MDA content of SLD48 was significantly lower than that of LD at 20‰–50‰ salt.

Notably, even under the extreme stress of 50‰ salt (where CAT activities were similar between the two strains), SLD48 still maintained significantly and consistently higher SOD activity, indicating that SOD serves as the core antioxidant enzyme for ROS scavenging in SLD48 under extreme salt stress.

To further investigate the improvements in osmotic adjustment and cell membrane integrity in the mutant strain SLD48, we determined the PRO content and electrical conductivity of LD and SLD48 under salt stress (Fig. 4d, e).

At tested salt concentrations 5‰, 20‰, and 50‰, the PRO content of SLD48 was significantly higher than that of LD. Both strains reached their peak PRO accumulation at 10‰ salt (LD: 0.7482 mg/g, SLD48: 0.7139 mg/g), and SLD48 showed a stronger ability to maintain PRO levels under high-salt conditions (Fig. 4d). Accordingly, the electrical conductivity of SLD48 was significantly lower than that of LD at all salt concentrations, and the difference became more pronounced with increasing salt concentration, indicating lower cell membrane permeability in SLD48 (Fig. 4e).

### Effects of Differential Metabolites on Alleviating Salt Stress

Based on metabolomic analysis, differential metabolites may play certain regulatory roles in alleviating salt stress. In this study, Pro, α-Toc, Bet, 5-AmA, and SuA were separately added under 8‰ NaCl stress, and the growth performance (OD₆₀₀ values and viable cell counts) of strains LD and SLD48 was determined, with blank control (CK) and salt stress control (NaCl) as references (Fig. 5).

The OD₆₀₀ results showed that SLD48 grew better than LD in both CK and salt stress treatments. After adding the above differential metabolites, the growth (OD₆₀₀) of LD was significantly upregulated compared with the salt stress control, but the effects on SLD48 were inconsistent. Pro, α-Toc, and Bet significantly enhanced the growth of SLD48 under salt stress, whereas 5-AmA showed no promoting effect, and SuA even inhibited the growth of SLD48 under salt stress (Fig. 5a).

The viable cell count results were different. Under salt stress, the viable cell counts of SLD48 remained unchanged compared with CK, while that of LD decreased significantly. After supplementation with the above differential metabolites, the viable cell counts of both LD and SLD48 increased significantly compared with the salt stress alone group (Fig. 5b).

Overall, Pro, α-Toc and Bet effectively alleviated the inhibitory effects of salt stress on the strains.

## Discussion

In this study, a high salt-tolerant *Bacillus megaterium* mutant SLD48 was obtained via atmospheric and room temperature plasma (ARTP) mutagenesis combined with microbial microdroplet culture (MMC) screening. Multi-omics data were further applied to dissect the potential salt-tolerant mechanisms of SLD48, and its plant growth-promoting capacity was validated through maize pot trials in saline–alkali soil. Our results provide novel insights into salt tolerance of *B. megaterium*.

As an efficient physical mutagenesis approach, ARTP has been successfully used to upgrade metabolic traits of *Methylosinus trichosporium* and increase lipid production of *Chlorella pyrenoidosa* in earlier reports [14, 18]. In the present work, obvious genomic variations were triggered in WT *B. megaterium* LD after ARTP treatment; the mutant SLD48 possessed an expanded genome and gained more functional genes, confirming that ARTP is capable of inducing genome-level genetic variation and serves as a reliable tool for microbial breeding. Unlike previous protocols adopting fixed single irradiation duration, we optimized the mutagenesis time at 25–35 s according to the optimal lethality of 85%–95% [4], which effectively increases the acquisition possibility of positive mutants. To our knowledge, this is the first report combining ARTP mutagenesis with dynamic salt-gradient screening based on the MMC platform, which enables precise isolation of mutants retaining high viability under 8.5‰ salinity and greatly improves screening efficiency and accuracy compared with conventional plate screening.

With respect to salt-resistance mechanisms, earlier studies have documented that plant-growth-promoting bacteria accumulate PRO and GB to maintain intracellular osmotic homeostasis, while α-Toc eliminates reactive oxygen species (ROS) to strengthen microbial environmental adaptability [4, 37]. Consistently, our metabolomic data revealed that PRO, GB and α-Toc contents in SLD48 rose by 515%, 403% and 497% relative to the WT LD, respectively. Moreover, exogenous supplementation of PRO, α-Toc and GB in medium markedly accelerated the growth of both LD and SLD48.

Existing researches on salt-tolerant genes of *B. megaterium* mainly focus on genes involved in polysaccharide synthesis and organic acid excretion. Herein, two unique genes were identified in SLD48: the RecA/Rad51 superfamily recombinase gene *spoIVCA* and MerR-family transcriptional regulator gene *pJ_gene0003*. Previous publications have proven their roles in DNA damage repair, heavy metal detoxification and horizontal gene transfer, implying their potential contributions to elevated salt tolerance of SLD48. Further gene-knockout assays are required to verify their exact biological functions in *B. megaterium*.

Numerous studies have verified the growth-promoting potential of *B. megaterium* via improving soil fertility and regulating rhizosphere microecology: certain strains ameliorate physiological and biochemical characteristics and enhance salt resistance of wheat [7], whereas strain NCT-2 optimizes nutrient status of secondarily salinized soil and elevates vegetable quality [9]. Our pot experiment data are in good agreement with these findings: SLD48 exhibited significantly higher rhizosphere colonization abundance than WT LD in saline–alkali soil, and maize shoot fresh weight was increased by 40.5% after SLD48 inoculation. These findings further validate the growth-promoting property of *B. megaterium* and broaden its application prospect for agricultural production in saline–alkali regions.

## Conclusion

In this study, the WT strain *Bacillus megaterium* LD was used as the original strain. A high salt-tolerant mutant SLD48 was successfully obtained via atmospheric and room-temperature plasma (ARTP) mutagenesis coupled with dynamic salinity-gradient screening using the microbial microdroplet culture (MMC) system. Through phenotypic characterization, combined genomic and metabolomic analysis, determination of physiological and biochemical indices, and pot experiments of maize in saline–alkali soil, this study clarified how SLD48 improves its own salt tolerance and plant growth-promoting potential through differential metabolites. The main conclusions are as follows:

Genomic analysis showed that the mutant strain SLD48 possessed a longer genome sequence, a higher number of plasmids and significantly increased functional genes. It contained unique functional genes such as *spoIVCA* and *pJ_gene0003*.

Metabolomic analysis confirmed that the contents of PRO, GB and α-Toc in SLD48 were substantially up-regulated.

Physiological and biochemical tests revealed that under high-salt conditions, SLD48 maintained higher activities of superoxide dismutase (SOD) and catalase (CAT), while reducing malondialdehyde (MDA) accumulation and cell membrane permeability. Exogenous addition of PRO, α-Toc and GB also effectively alleviated the growth inhibition of strains caused by salt stress, which verified the salt-tolerant function of key metabolites.

Pot experiments in saline–alkali soil indicated that the rhizosphere colonization ability of mutant strain SLD48 was much higher than that of the WT strain. It could significantly promote maize growth, with the shoot fresh weight increased by 40.5%, exhibiting excellent rhizosphere colonization capacity and plant growth-promoting effect.

## Supplementary Information

Below is the link to the electronic supplementary material.

Supplementary Material 1

Supplementary Material 2: Salt tolerance evaluation of WT strain. **a** Lethality curve of WT strain with ARTP mutagenesis, **b** Salt-tolerant growth of WT strain under different salt concentrations

Supplementary Material 3: The morphological characteristics of LD and SLD48 under stereomicroscope and scanning electron microscope (SEM). **a** stereomicroscope of LD, **b** stereomicroscope of SLD48, **c** scanning electron microscope (SEM) of LD, **d** scanning electron microscope (SEM) of SLD48. The scale bar represents 1 mm in **a** and **b**, the scale bar represents 2μm in **c** and **d**

Supplementary Material 4: Growth curves of LD and SLD48 under different salt concentrations. **a** viable cell counts of LD within 48 hours; **b** viable cell counts of SLD48 within 48 hours

## Data Availability

No datasets were generated or analysed during the current study.
