# Supplementary material for: A Novel ARTP-derived Bacillus megaterium Mutant with Enhanced Salt Tolerance and Plant Growth Promotion in Saline–alkali Soil
Source: Curr Microbiol. 2026 Aug 2;83(9):502. doi: 10.1007/s00284-026-05077-9 (PMC13429540; doi:10.1007/s00284-026-05077-9)
Supplement: Supplementary file 1 — Supplementary Material 1 [file 284_2026_5077_MOESM1_ESM.docx]

**Sup table 1 Basic physicochemical properties of the tested alkaline soil**

| **pH** | **EC**  **(HS/cm)** | **Organic matter (g/kg)** | **Alkali-hydrolyzable nitrogen (mg/kg)** | **Available phosphorus (mg/kg)** | **Available potassium (mg/kg)** | **Exchangeable calcium (mg/kg)** | **Exchangeable sodium (mg/kg)** | **Exchangeable magnesium (mg/kg)** | **Salt content (g/kg)** |
| --- | --- | --- | --- | --- | --- | --- | --- | --- | --- |
| **9.0** | **1794** | **5.73** | **35.5** | **8.9** | **100** | **3914.9** | **1873.1** | **425.2** | **5.60** |
