# Supplementary figures and images for: A Novel ARTP-derived Bacillus megaterium Mutant with Enhanced Salt Tolerance and Plant Growth Promotion in Saline–alkali Soil
Source: Curr Microbiol. 2026 Aug 2;83(9):502. doi: 10.1007/s00284-026-05077-9 (PMC13429540; doi:10.1007/s00284-026-05077-9)

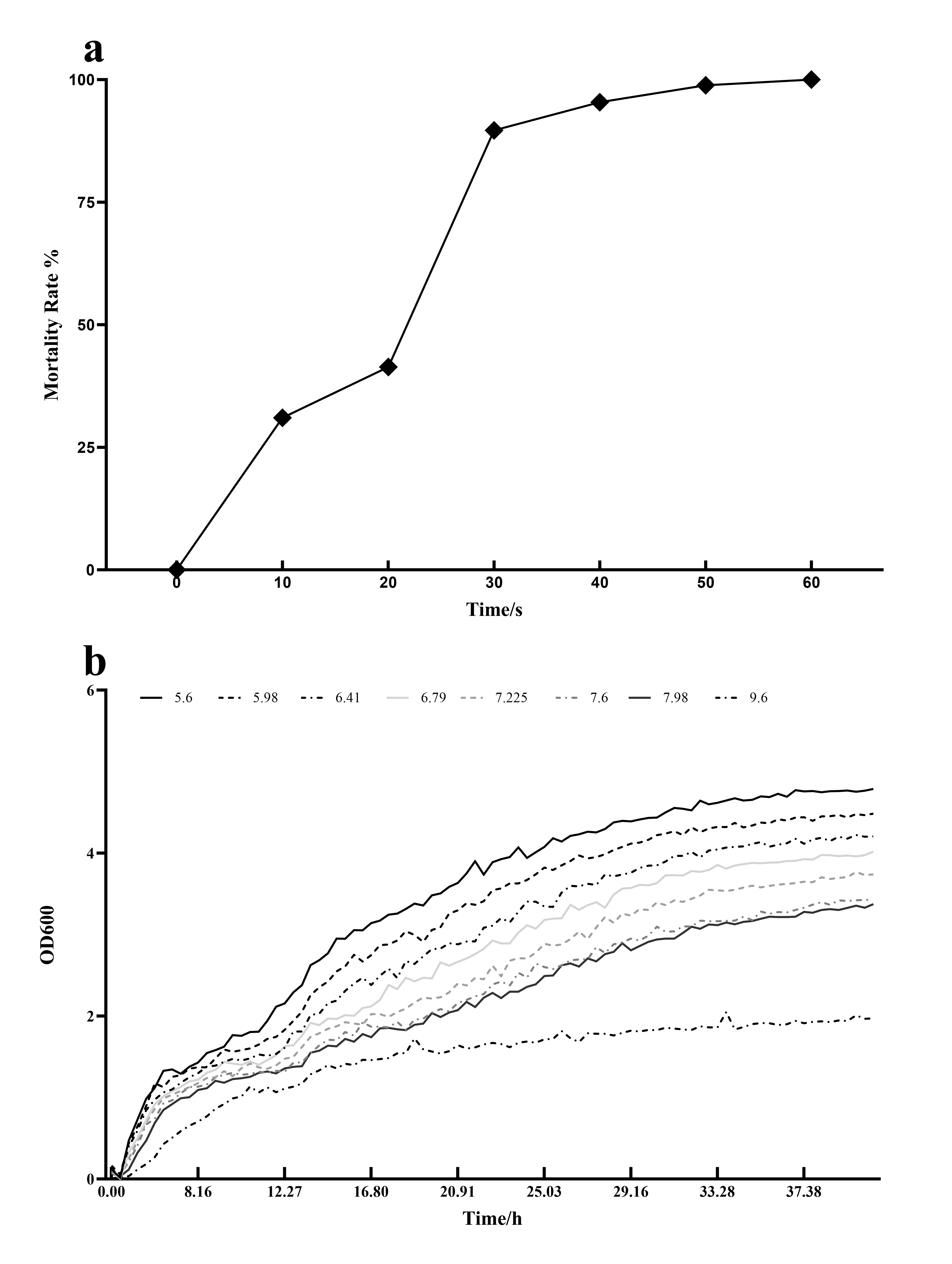

Supplement: Supplementary file 2 — Supplementary Material 2: Salt tolerance evaluation of WT strain. a Lethality curve of WT strain with ARTP mutagenesis, b Salt-tolerant growth of WT strain under different salt concentrations [file 284_2026_5077_MOESM2_ESM.tif]

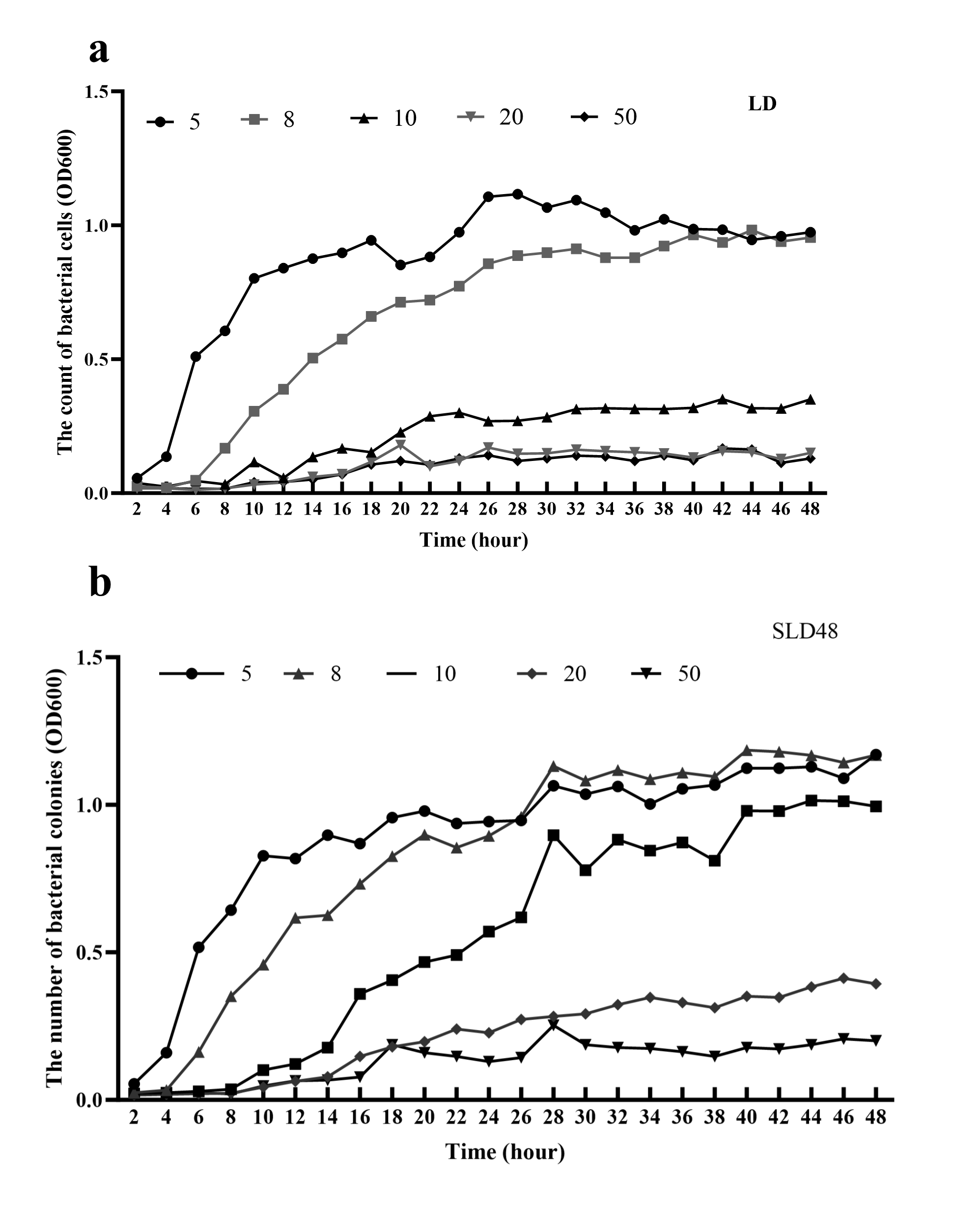

Supplement: Supplementary file 4 — Supplementary Material 4: Growth curves of LD and SLD48 under different salt concentrations. a viable cell counts of LD within 48 hours; b viable cell counts of SLD48 within 48 hours [file 284_2026_5077_MOESM4_ESM.tif]
